# Risk of retinal vascular occlusion following COVID-19 vaccination: A meta-analysis of nearly 15 million individuals

**DOI:** 10.1097/MD.0000000000051345

**Published:** 2026-11-06

**Authors:** Omar Khalid Samir, Noheir Ashraf Ibrahem Fathy Hassan, Raymond Haward, Ishaan Batish, Amr Arafa, Eslam Alwani Haiba, Mahmoud Aboudabash, M. Khodr Aljamali, Mohamed AlMadhoob, Saad Yousafzai, Yusef Hazimeh, Eman A. Toraih, Hani Aiash

**Affiliations:** a Faculty of Medicine, Port Said University, Port Said, Egypt; b Faculty of Medicine, Aswan University, Aswan, Egypt; c Department of Internal Medicine, Vydehi Institute of Medical Sciences and Research Centre, Bangalore, Karnataka, India; d Department of Internal Medicine, Sri Guru Ram Das Institute of Medical Sciences and Research, Amritsar, Punjab, India; e Faculty of Medicine, Alexandria University, Alexandria, Egypt; f School of Medicine, New Giza University, Giza, Egypt; g IM Department, South Texas Health System, McAllen, Texas; h School of Medicine, Royal College of Surgeons in Ireland, Dublin, Ireland; i Department of Internal Medicine, Division of Endocrinology, American University of Beirut, Beirut, Lebanon; j Faculty of Medicine, Suez Canal University, Ismailia, Egypt; k Cardiovascular Perfusion Department, SUNY Upstate Medical University, Syracuse, New York; l Simulation Center, SUNY Upstate Medical University, Syracuse, New York.

**Keywords:** COVID-19 vaccine, RAO, retinal artery occlusion, retinal vascular occlusion, retinal vein occlusion, RVO

## Abstract

**Background:**

Retinal vascular occlusion is a rare ocular condition reported following coronavirus disease 2019 (COVID-19) vaccination. However, the association between vaccination and retinal vascular occlusion remains uncertain. This systematic review and meta-analysis evaluated the frequency and risk of retinal vascular occlusion following COVID-19 vaccination.

**Methods:**

Four electronic databases were systematically searched through August 2026. Observational studies reporting retinal vascular occlusion after COVID-19 vaccination were included. Random-effects meta-analyses were performed to estimate pooled event proportions and risk ratios (RRs) with 95% confidence intervals (CIs). Potentially overlapping study populations were assessed, and study quality was evaluated using the Newcastle–Ottawa Scale.

**Results:**

Five studies comprising 14,877,781 reported participants were included. The pooled proportion of retinal vascular occlusion events among vaccinated individuals was 0.02% (95% CI: 0.00%–0.07%; *I*^2^ = 99.5%). The overall association between vaccination and retinal vascular occlusion was not statistically significant (RR = 2.12, 95% CI: 0.65–6.90; *P* = .213), with substantial heterogeneity (*I*^2^ = 96.8%). However, separate analyses showed statistically significant associations for retinal artery occlusion (RR = 2.03, 95% CI: 1.17–3.51) and retinal vein occlusion (RR = 2.26, 95% CI: 2.08–2.47), without a significant difference between subtypes (*P* = .696). No significant associations between retinal vascular occlusion and vaccine were observed in sex-specific analyses. Among vaccine types, ChAdOx1/AstraZeneca showed a significant association (RR = 2.00, 95% CI: 1.60–2.50), based on a single study, whereas pooled associations for other vaccines were nonsignificant.

**Conclusion:**

Retinal vascular occlusion events following COVID-19 vaccination were rare. Although the overall association was not statistically significant, separate arterial and venous analyses showed significant associations. Substantial heterogeneity, potential confounding, and differences in contributing studies limit definitive conclusions. Further research is needed to clarify these findings.

## 1. Introduction

There have been 777 million cases of coronavirus disease 2019 (COVID-19) as of June 2025, with an estimated 7 million fatalities.^[1]^ Numerous vaccines, such as adjuvanted recombinant protein vaccines (Novavax), mRNA vaccines (BNT162b2/Pfizer-BioNTech and mRNA-1273/Moderna), and adenoviral-vector vaccines (ChAdOx1/AstraZeneca and Ad26.COV2.S/Janssen) have been developed since the end of 2020. As a result, higher vaccination rates have been linked to several potential issues.^[2]^ The effectiveness of COVID-19 vaccinations in reducing hospitalization rates and mortality has been demonstrated.^[3,4]^ Vaccines have been given in 11.9 billion doses worldwide so far.^[1]^

According to a neurologist’s survey, the severe acute respiratory syndrome coronavirus 2 immunization increased the incidence of cerebral sinus and venous thromboses.^[5]^ At the same time, some issues in the anterior and posterior eye segments were reported in ophthalmological case reports and short case series.^[6–8]^ Retinal vein occlusion (RVO) is the 2nd most common cause of vision loss related to retinal blood vessel problems, right after diabetic retinopathy. RVO is linked to thromboembolism caused by vascular wall deterioration, arterial compression, or vasospasm.^[9]^ Retinal artery occlusion (RAO) happens when the retinal arteries from the same side’s carotid artery, aortic arch, or heart get blocked or narrowed due to spasms, inflammation, poor blood flow, or blood clots. RAO and RVO can be classified into central or branch types based on where the blockage happens. Infection with severe acute respiratory syndrome coronavirus 2 may lead to problems with the retinal blood vessels.^[10,11]^ Retinal vascular occlusion after receiving different types of COVID-19 vaccines is quite rare. However, a growing body of evidence of occlusion after immunization includes case reports.^[12–21]^ Interestingly, some studies have linked the mRNA COVID-19 vaccine to retinal vascular occlusion.^[22,23]^ However, they were not enough to prove that the COVID-19 vaccine and retinal vascular occlusion were causally related.

The licensing procedures for adequate vaccinations have been different from those for other medication approvals because of the pressing demand for suitable vaccines against COVID-19. Because of this, the prevalence of serious adverse events may be overestimated or underestimated when using drugs outside of scientific trials, and side effects after vaccination may not be completely recorded. Because of this vagueness, doctors find it challenging to advise patients about possible vaccination-related adverse effects.^[24,25]^

We carried out this systematic review and meta-analysis to evaluate the relationship between the risk of retinal vascular occlusion and COVID-19 immunization to fill this gap. Our objectives were to: estimate the frequency of occlusions following COVID-19 vaccination compared to unvaccinated controls, evaluate the relationship between retinal vascular occlusions and COVID-19 vaccinations, and evaluate the association between occlusions and different types of vaccines.

## 2. Methods

This systematic review and meta-analysis were conducted strictly following the Preferred Reporting Items for Systematic Reviews and Meta-Analyses guidelines.^[26]^ The study protocol has been registered with International Prospective Register of Systematic Reviews under the registration number CRD420251038822.

### 2.1. Ethical review

No ethical approval was required for this study, as it is a systematic review and meta-analysis of already published studies.

### 2.2. Search strategy

We searched 4 major electronic databases (PubMed, Medline, Web of Science, and Scopus) up to August 2026, using a search strategy tailored to each database. The search strategy combined keywords about different types of COVID-19 vaccines and different types of retinal vascular occlusion. The search details can be found in Table S1, Supplemental Digital Content 1.

### 2.3. Eligibility criteria

During the screening stage, at least 2 reviewers separately screened the articles. We included observational studies that: reported cases of retinal vascular occlusion after receiving any type of the COVID-19 vaccines; included participants of any age; provided information on the number of vaccinated and unvaccinated participants where applicable; reported at least 1 vascular occlusion outcome (RVO, RAO, central RAO, branch RAO, central RVO, or branch RVO); and were published in English.

We excluded any studies that: were editorials, commentaries, or conference abstracts; did not provide information about vaccination status; focused exclusively on COVID-19 infection rather than vaccination.

### 2.4. Data extraction

Two reviewers separately extracted the data using a standardized form, and any disagreements were discussed with a 3rd reviewer. We collected available data from the included studies on: study characteristics: last name of the 1st author, publication year, country, study design, follow-up duration, and types of vaccines examined; population characteristics: sample size, demographics (age and sex); retinal vascular occlusion outcomes: (RAO, RVO, central RAO, branch RAO, central RVO, and branch RVO) in both vaccinated and unvaccinated groups; and vaccination details.

### 2.5. Quality assessment

To evaluate the included studies’ quality, we used the Newcastle–Ottawa Scale (NOS).^[27]^ The scale assessed the studies based on the selection of study groups, comparability of groups, and ascertainment of exposure or outcome.

Two reviewers independently evaluated the studies. A 3rd reviewer was consulted when conflicts arose. The studies were classified as high quality if they scored 7 or above on the NOS, moderate quality if they scored between 5 and 6, and low quality if they scored 4 or below.

### 2.6. Statistical analysis

In the current study, we displayed continuous data as mean and standard deviation and dichotomous data as frequency and percentage (%). We calculated risk ratios (RRs) with 95% confidence intervals (CIs) to compare the likelihood of retinal vascular occlusion between vaccinated and unvaccinated groups. Risk ratios were calculated from raw event counts and denominators rather than pooled adjusted estimates.

We employed random-effects models using the restricted maximum likelihood method for all meta-analyses due to heterogeneity. For event proportions, we used logit-transformed inverse variance. We tested heterogeneity using the *I*^2^ statistic. Publication bias assessment was planned but not performed, as the number of included studies was <10. We also applied the chi-squared test to decide whether the heterogeneity was statistically significant. The analyses were performed in R version 4.2.0 (RStudio 2025.05.0 Build 496), using the “meta” package.

Potential overlap between studies was assessed by comparing data sources, study periods, populations, eligibility criteria, and outcomes. Kim et al and Park et al used overlapping South Korean national health databases.^[28,29]^ Kim et al was prioritized for comparable analyses because it directly assessed overall retinal vascular occlusion using age- and sex-matched sampling and a defined 60-day follow-up. Park et al was retained for outcome-specific analyses, including separate RAO and RVO outcomes, where the required data were unavailable from Kim et al.

Similarly, Dorney et al and Li et al used TriNetX data with overlapping observation periods.^[30,31]^ Dorney et al was retained for the pooled event-proportion analysis because it provided clearly defined event counts and denominators within a 21-day follow-up. Li et al was retained for other analyses involving comparisons unavailable from Dorney et al The extent of individual-level overlap could not be determined from the published data. Selection decisions were based on methodological comparability and outcome availability rather than statistical significance.

## 3. Results

### 3.1. Study selection and baseline characteristics

We searched 4 major databases using tailored search strategies, and 1365 records were identified. After screening, 5 eligible studies were included in our study^[28–32]^ (Fig. 1). These studies collectively included 14,877,781 participants (11,341,856 vaccinated individuals and 3,535,925 unvaccinated controls). Studies were published between 2022 and 2024. Sample sizes varied substantially, ranging from 172,366^[32]^ to 7,841,119^[29]^ participants. The mean age of the studied individuals was 55.42 ± 17.96, with no significant age difference observed between the vaccinated and unvaccinated (mean difference: 5.83 years, 95% CI: −3.94 to 15.59). Additional characteristics of the included studies are summarized in Table 1.

**Table 1:** Characteristics of included studies.

Author (year)	Country	Study design	Follow-up period	Vaccine type(s)	Sample size			Mean age ± SD	Female (%)	Ref.
					Case	Control	Total			
Park (2024)	South Korea	Nationwide population-based retrospective cohort	60 d	1. ChadOx1/AstraZeneca 2. BNT162b2/BioNTech 3. mRNA-1273/Moderna 4. Ad26.COV2.S/Johnson & Johnson	5,160,681	2,680,438	7,841,119	47.12 ± 15.8	68.2	[29]
Kim (2024)	South Korea	Retrospective Cohort	60 d	1. ChAdOx1/AstraZeneca 2. BNT162b2/BioNTech 3. mRNA-1273/Moderna 4. Ad26.COV2.S/Johnson & Johnson	2,479,462	262,603	2,742,065	48.68 ± 17.13	53.9	[28]
Li (2023)	Taiwan	Retrospective cohort study with propensity score matching (US collaborative network, TriNetX)	2 yr	1. BNT162b2/BioNTech 2. mRNA-1273/Moderna 3. Ad26.COV2.S/Johnson & Johnson	506,701	506,701	1,013,402	52.35 ± 18.35	–	[30]
Dorney (2023)	USA	Retrospective cohort study with propensity score matching (the COVID-19 research network, TriNetX)	21 d	mRNA-1273/Moderna	3,108,829	–	3,108,829	50.7 ± 20.4	56.4	[31]
Hashimoto (2022)	Japan	Matched cohort and self-controlled case series (SCCS) studies	84 d	BNT162b2/BioNTech	86,183	86,183	172,366	69.3 ± 16.6	78.2	[32]

Dashes (–) indicate data not available or not reported in the original study.

Ref. = Reference, US = United States of America.

**Figure 1.**
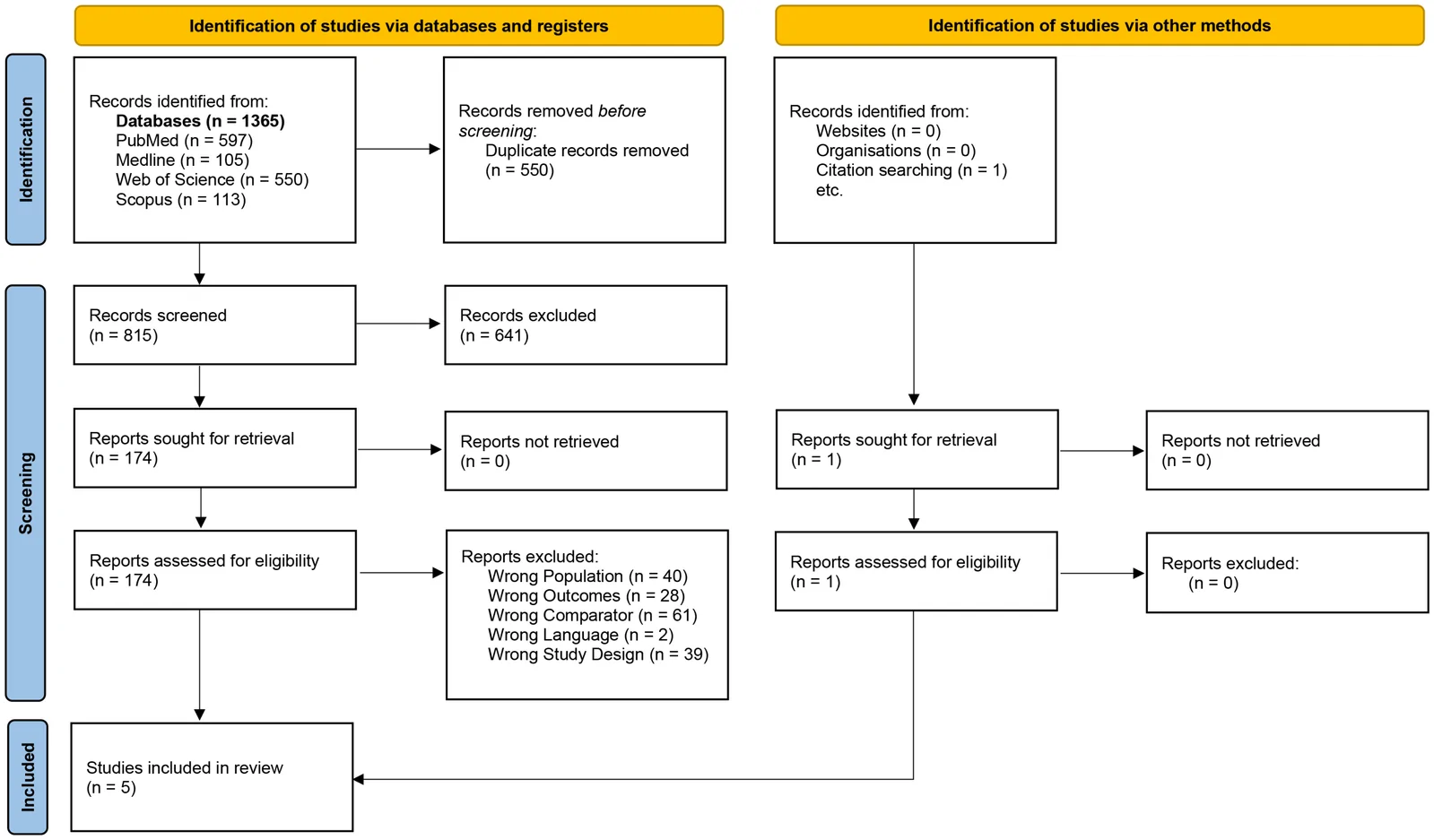
PRISMA flow diagram.^[26]^ PRISMA = Preferred Reporting Items for Systematic Reviews and Meta-Analyses.

### 3.2. Quality assessment

The NOS scores were between 5 and 8. Four studies were of high quality,^[28,29,31,32]^ while only 1 study was of moderate quality.^[30]^ No studies were of low quality (Table S2, Supplemental Digital Content 2).

### 3.3. Retinal vascular occlusion (overall)

The pooled proportion of retinal vascular occlusion events among vaccinated individuals included 859 events among 5,674,474 vaccinated individuals across 3 studies and was 0.02% (95% CI: 0.00%–0.07%), with considerable heterogeneity (*I*^2^ = 99.5%, *P* < .0001) (Fig. S1, Supplemental Digital Content 3). Our overall pooled analysis showed no statistically significant association between COVID-19 vaccination and retinal vascular occlusion (RR = 2.12, 95% CI: 0.65–6.90, *P* = .213). Substantial heterogeneity was present between studies (*I*^2^ = 96.8%, *P* < .0001) (Fig. 2).

**Figure 2.**
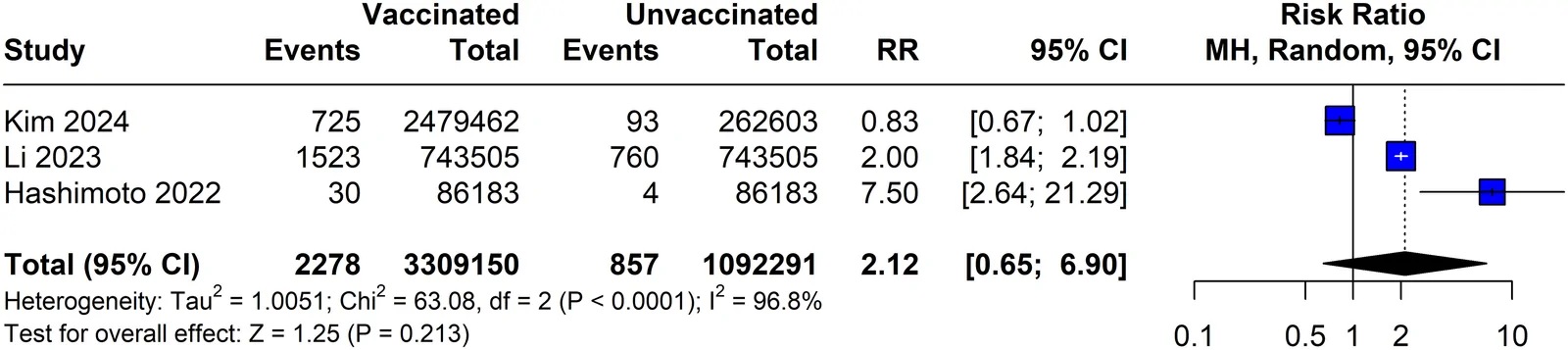
Forest plot of retinal vascular occlusion risk in vaccinated versus unvaccinated individuals. Data are presented as risk ratios (RRs) and 95% confidence intervals (CIs). MH = Mantel–Haenszel.

#### 3.3.1. Follow-up subgroups

A subgroup analysis according to follow-up duration (1, 2, 3, and 24 months) was conducted to explore differences in retinal vascular occlusion risk across observation periods (Fig. 3). In the 1-month subgroup, the pooled association was not statistically significant (RR = 1.29, 95% CI: 0.64–2.59, *P* = .469), with moderate heterogeneity (*I*^2^ = 40.7%). The 2-month subgroup, represented by a single study, also showed no significant association (RR = 0.83, 95% CI: 0.67–1.02). The 3-month subgroup demonstrated the highest pooled point estimate, although the association remained statistically nonsignificant (RR = 2.94, 95% CI: 0.55–15.83, *P* = .209), with substantial heterogeneity (*I*^2^ = 89.5%, *P* = .002). The 24-month subgroup was represented by a single study reporting an RR of 1.21 (95% CI: 1.00–1.46). The test for subgroup differences was statistically significant (*P* = .035), suggesting variation in the estimated associations across follow-up durations. However, these findings should be interpreted cautiously given the limited number of studies within individual subgroups and substantial between-study heterogeneity.

**Figure 3.**
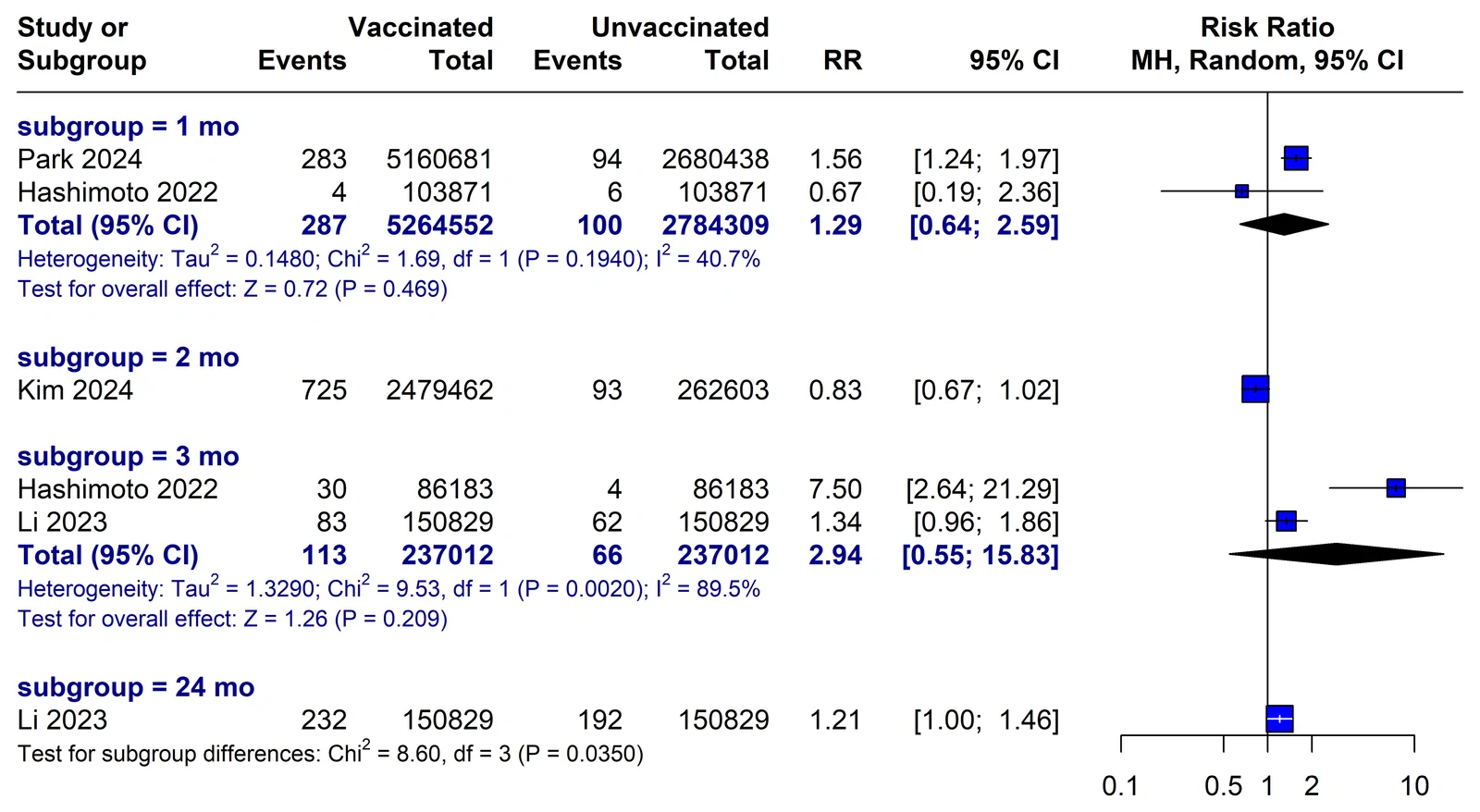
Forest plot of retinal vascular occlusion risk in vaccinated versus unvaccinated individuals stratified by follow-up duration Data are presented as risk ratios (RRs) and 95% confidence intervals (CIs). MH = Mantel–Haenszel.

#### 3.3.2. Sex subgroups

No statistically significant association was observed between COVID-19 vaccination and retinal vascular occlusion among males (RR = 1.26, 95% CI: 0.40–3.92, *P* = .691), with substantial heterogeneity (*I*^2^ = 97.9%). Similarly, the female subgroup showed no significant association (RR = 1.47, 95% CI: 0.67–3.25, *P* = .337), with high heterogeneity (*I*^2^ = 95.5%). The test for subgroup differences was not statistically significant (*P* = .824), indicating no evidence of a difference between males and females (Fig. S2, Supplemental Digital Content 4).

#### 3.3.3. Age subgroups (<65 vs ≥65 years)

In the <65 years age subgroup, there was no significant difference in the risk of retinal vascular occlusion between vaccinated and unvaccinated individuals (RR = 1.23, 95% CI: 0.62–2.46). Similarly, there was no significant difference in the ≥65 years age subgroup (RR = 1.27, 95% CI: 0.45–3.58). The subgroup difference was insignificant (*P* = .96), suggesting no significant difference between the age groups (Fig. S3, Supplemental Digital Content 5).

### 3.4. Retinal artery occlusion (RAO)

The risk of RAO was significantly associated with vaccination with different types of COVID-19 vaccines in comparison with the unvaccinated controls, according to the pooled analysis (RR = 2.03, 95% CI: 1.17–3.51, *P* = .012) (Fig. 4).

**Figure 4.**
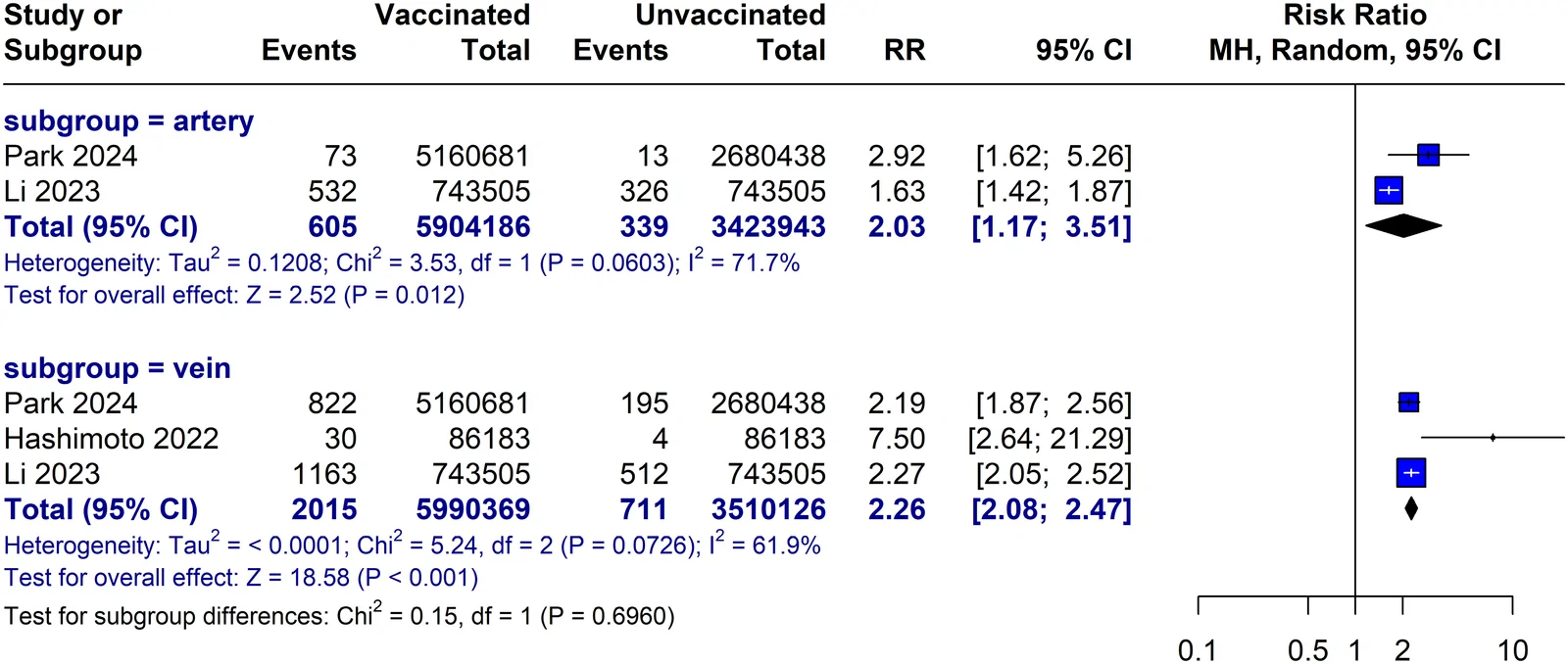
Forest plot of retinal artery occlusion (RAO) and retinal vein occlusion (RVO) risk in vaccinated versus unvaccinated individuals. Data are presented as risk ratios (RRs) and 95% confidence intervals (CIs). MH = Mantel–Haenszel.

#### 3.4.1. Sex subgroups

In the male subgroup, vaccination was significantly associated with a higher risk of retinal artery occlusions (RR = 1.91, 95% CI: 1.45–2.52). Similarly, a significant association was found among females (RR = 2.06, 95% CI: 1.19–3.57). The subgroup difference was insignificant (*P* = .807), suggesting no significant difference between both sexes (Fig. S4, Supplemental Digital Content 6).

### 3.5. Retinal vein occlusion (RVO)

The risk of RVO was significantly associated with being COVID-19 vaccinated in comparison with the unvaccinated controls, according to the pooled analysis (RR = 2.26, 95% CI: 2.08–2.47, *P* < .001). Notably, this risk was higher than RAO’s (Fig. 4).

#### 3.5.1. Sex subgroups

In the male subgroup, the risk of RVO was significantly associated with vaccination compared to the unvaccinated controls (RR = 2.26, 95% CI: 1.69–3.04). Similarly, a significant association was found in the female subgroup (RR = 2.46, 95% CI: 2.17–2.79). The subgroup difference was insignificant (*P* = .609), suggesting no significant difference between both sexes (Fig. S5, Supplemental Digital Content 7).

### 3.6. Vaccines

The pooled proportion of retinal vascular occlusion events was evaluated across 4 COVID-19 vaccines: ChAdOx1/AstraZeneca, BNT162b2/Pfizer-BioNTech, mRNA-1273/Moderna, and Ad26.COV2.S/Janssen (Fig. S6, Supplemental Digital Content 8). The event proportion following ChAdOx1/AstraZeneca vaccination was 0.07% (95% CI: 0.06%–0.08%), based on a single study. The pooled event proportions were 0.04% (95% CI: 0.01%–0.14%) for BNT162b2/Pfizer-BioNTech, 0.05% (95% CI: 0.00%–0.93%) for mRNA-1273/Moderna, and 0.05% (95% CI: 0.01%–0.37%) for Ad26.COV2.S/Janssen. Substantial heterogeneity was observed in these pooled subgroups (*I*^2^ = 99.4%, 99.6%, and 96.1%, respectively). The test for subgroup differences was not statistically significant (*P* = .778).

#### 3.6.1. Vaccine-type subgroups

The pooled analysis showed no statistically significant association between BNT162b2/Pfizer-BioNTech vaccination and retinal vascular occlusion (RR = 1.34, 95% CI: 0.24–7.29), with substantial heterogeneity (*I*^2^ = 96.5%). Similarly, no significant associations were observed for mRNA-1273/Moderna (RR = 0.67, 95% CI: 0.16–2.90, *I*^2^ = 97.5%) or Ad26.COV2.S/Janssen (RR = 0.65, 95% CI: 0.35–1.22, *I*^2^ = 39.0%). In contrast, ChAdOx1/AstraZeneca showed a statistically significant association with retinal vascular occlusion (RR = 2.00, 95% CI: 1.60–2.50), although this estimate was derived from a single study. The test for subgroup differences was statistically significant (*P* = .0055) (Fig. 5). These findings should be interpreted cautiously given the limited number of contributing studies, substantial heterogeneity, and potential differences in vaccine allocation and baseline population characteristics.

**Figure 5.**
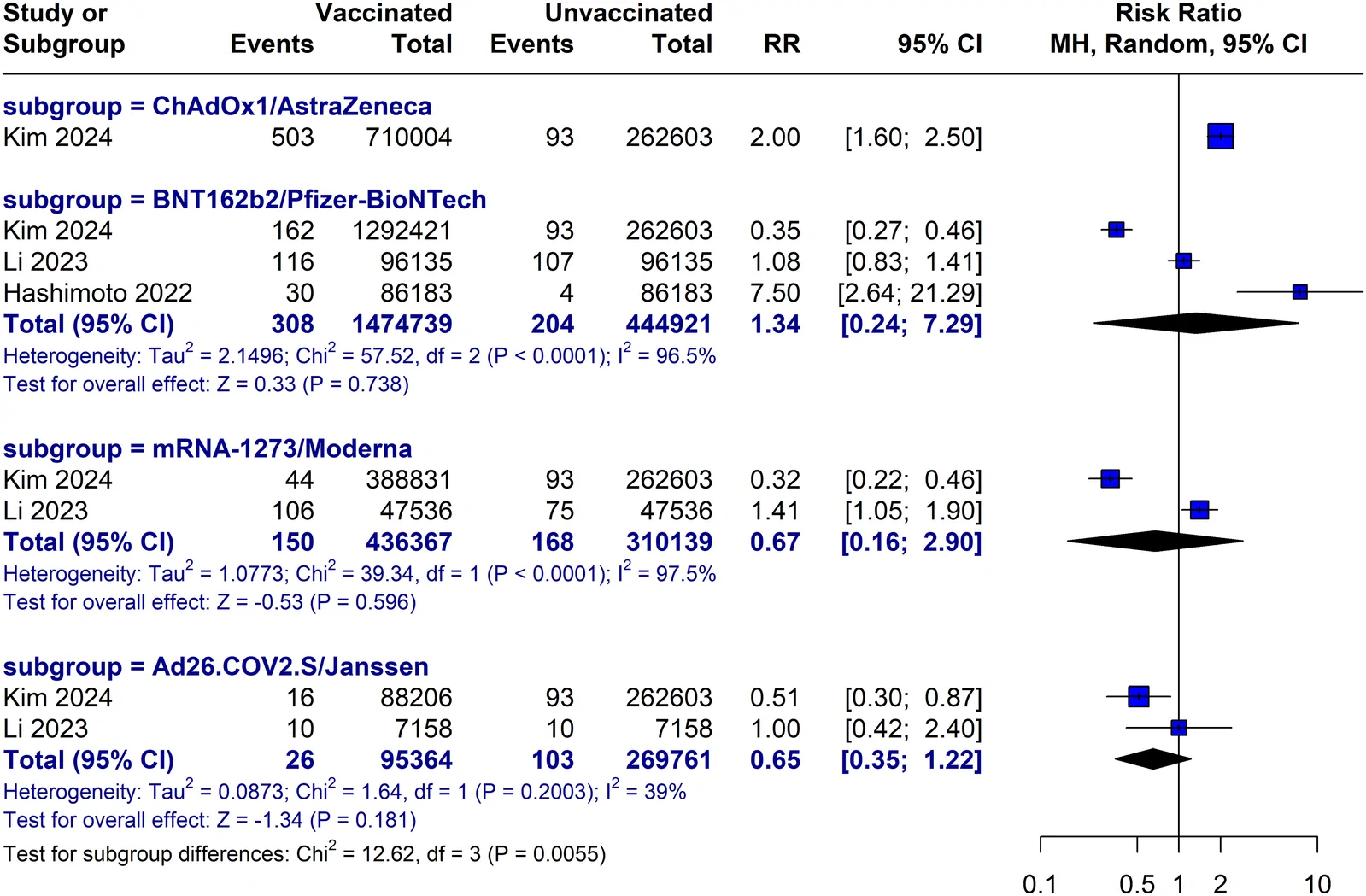
Forest plot of retinal vascular occlusion risk in vaccinated versus unvaccinated individuals stratified by vaccine types. Data are presented as risk ratios (RRs) and 95% confidence intervals (CIs). MH = Mantel–Haenszel.

#### 3.6.2. Dose subgroups (1st vs 2nd)

There was no subgroup difference between the st and the 2nd dose regarding retinal vascular occlusion risk (*P* = .27) (Fig. S7, Supplemental Digital Content 9).

#### 3.6.3. Vaccine type and follow-up time

Retinal vascular occlusion risk was assessed across 3 vaccine types by follow-up duration (Fig. 6). For BNT162b2/Pfizer-BioNTech, risk was significantly lower at 2 months (RR = 0.35, 95% CI: 0.27–0.46), but not at 3 months (RR = 3.01, 95% CI: 0.58–15.74; *I*^2^ = 88.0%) or 24 months (RR = 1.08, 95% CI: 0.83–1.41). Subgroup differences were significant (*P* < .0001) (Fig. 6A). For Ad26.COV2.S/Janssen, risk was significantly lower at 2 months (RR = 0.51, 95% CI: 0.30–0.87), with no significant associations at 3 or 24 months (Fig. 6B). For mRNA-1273/Moderna, risk was significantly lower at 2 months (RR = 0.32, 95% CI: 0.22–0.46), nonsignificant at 3 months (RR = 1.43, 95% CI: 0.84–2.44), and significantly higher at 24 months (RR = 1.41, 95% CI: 1.05–1.90). Subgroup differences were significant (*P* < .0001) (Fig. 6C).

**Figure 6.**
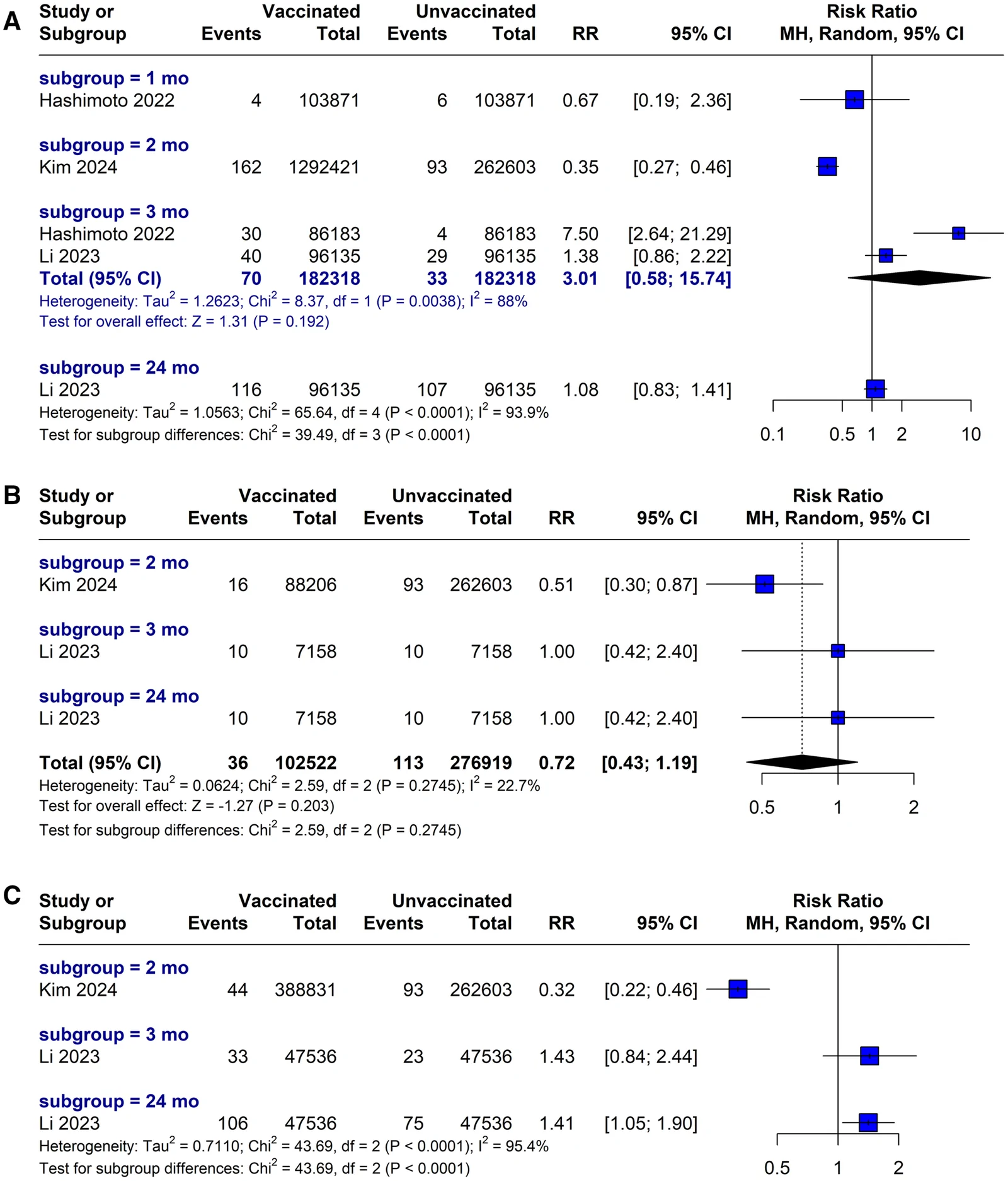
Forest plot of retinal vascular occlusion risk in vaccinated versus unvaccinated individuals stratified by follow-up duration. (A) = BNT162b2/Pfizer-BioNTech; (B) = Ad26.COV2.S/Janssen; and (C) = mRNA-1273/Moderna. Data are presented as risk ratios (RRs) and 95% confidence intervals (CIs). MH = Mantel–Haenszel.

### 3.7. Heterogeneity analysis

Substantial heterogeneity was observed across several analyses, including the overall retinal vascular occlusion risk (*I*^2^ = 96.8%) and pooled event proportion (*I*^2^ = 99.5%). High heterogeneity was also detected in the male (*I*^2^ = 97.9%) and female (*I*^2^ = 95.5%) subgroups. In the vaccine-type risk analyses, heterogeneity remained substantial for BNT162b2/Pfizer-BioNTech (*I*^2^ = 96.5%) and mRNA-1273/Moderna (*I*^2^ = 97.5%), while the Ad26.COV2.S/Janssen subgroup showed lower heterogeneity (*I*^2^ = 39.0%). The vaccine-specific event-proportion analyses also demonstrated substantial heterogeneity, with *I*^2^ values exceeding 95% across the pooled vaccine subgroups. These differences may reflect variations in study populations, vaccine types, observation periods, and outcome ascertainment, requiring cautious interpretation of the pooled estimates.

## 4. Discussion

The current meta-analysis evaluated the association between COVID-19 vaccination and retinal vascular occlusion using data from 5 observational studies. The pooled proportion of retinal vascular occlusion events among vaccinated individuals was very low (0.02%). Following the exclusion of potentially overlapping study populations, the overall analysis showed no statistically significant association between COVID-19 vaccination and retinal vascular occlusion (RR = 2.12, 95% CI: 0.65–6.90). However, substantial heterogeneity (*I*^2^ = 96.8%) and the wide CI indicate considerable uncertainty in the pooled estimate. Although the vaccine-type subgroup analysis suggested differences between vaccine products, these findings were based on limited evidence and should be interpreted cautiously.

Interestingly, although the overall pooled analysis did not demonstrate a statistically significant association between COVID-19 vaccination and retinal vascular occlusion, the outcome-specific analyses showed significant associations for both RAO (RAO; RR = 2.03, 95% CI: 1.17–3.51) and RVO (RVO; RR = 2.26, 95% CI: 2.08–2.47). This discrepancy may partly reflect differences in the studies contributing to each analysis, particularly the inclusion of Kim et al in the overall analysis but not in the artery- and vein-specific analyses. Kim et al reported a lower overall risk among vaccinated individuals, which may have influenced the overall pooled estimate. However, the absence of a statistically significant difference between the RAO and RVO subgroups (*P* = .696) does not support a stronger association with either vascular occlusion subtype. Furthermore, substantial heterogeneity remained in both analyses (*I*^2^ = 71.7% and 61.9%, respectively), and the findings should be interpreted cautiously given the differences in contributing study populations and the potential for residual confounding.

### 4.1. Comparison with previous literature

Current literature presents conflicting evidence regarding COVID-19 vaccination and retinal vascular occlusion risk. Kim et al, analyzing >2.7 million individuals in South Korea, reported a lower risk of retinal vascular occlusion following vaccination, although this finding may reflect differences in baseline characteristics or residual confounding.^[28]^ Similarly, Dorney et al found no elevated risk following mRNA vaccination,^[30]^ while Yeo et al concluded that existing case reports lack causality due to the absence of control groups.^[33]^

In contrast, Li et al reported an increased risk of retinal vascular occlusion following COVID-19 vaccination, particularly over longer follow-up periods.^[31]^ These conflicting findings may reflect differences in study populations, vaccine exposures, outcome ascertainment, and observation periods. Our pooled analysis did not demonstrate a statistically significant overall association, although substantial heterogeneity and imprecise estimates limit definitive conclusions.

Notably, the follow-up subgroup analysis revealed statistically significant differences across observation periods (*P* = .035). However, several subgroups were represented by individual studies, and the observed differences may reflect between-study methodological variations rather than a consistent temporal association. Multiple case reports have also documented temporal associations.^[13,14,19,20]^

### 4.2. Potential mechanisms and clinical implications

Retinal vascular occlusion is a multifactorial condition involving vascular and systemic risk factors, with differing mechanisms underlying arterial and venous occlusions.^[9]^ In the context of COVID-19 vaccination, immune-mediated thrombosis and inflammatory responses have been discussed as potential biological considerations. Vaccine-induced immune thrombotic thrombocytopenia, characterized by platelet activation and thrombosis, has been documented following certain adenoviral-vector vaccines.^[34,35]^ However, the studies included in our meta-analysis did not provide platelet counts, anti-PF4 antibody measurements, or other mechanistic evidence linking vaccine-induced immune thrombotic thrombocytopenia to retinal vascular occlusion. Moreover, inflammatory or thrombotic mechanisms cannot be inferred from epidemiological associations alone. Therefore, although existing literature provides biological context for investigating potential vaccine-associated vascular events, our findings do not identify a specific mechanism or establish a causal relationship between COVID-19 vaccination and retinal vascular occlusion. Our vaccine-type subgroup analysis identified a statistically significant association with ChAdOx1/AstraZeneca (RR = 2.00, 95% CI: 1.60–2.50), although this estimate was derived from a single study. No statistically significant pooled associations were observed for BNT162b2/Pfizer-BioNTech, mRNA-1273/Moderna, or Ad26.COV2.S/Janssen. Although the test for subgroup differences was significant (*P* = .0055), differences in vaccine allocation, baseline population characteristics, follow-up periods, and multiple subgroup comparisons limit the reliability of vaccine-specific conclusions.

The sex-stratified analyses showed no statistically significant association between vaccination and retinal vascular occlusion in either males (RR = 1.26, 95% CI: 0.40–3.92) or females (RR = 1.47, 95% CI: 0.67–3.25). Furthermore, the test for subgroup differences was not significant (*P* = .824), providing no evidence of a sex-specific difference in the pooled associations.

While retinal vascular occlusion events were rare, with a pooled proportion of 0.02%, this estimate reflects events recorded during different observation periods and should not be interpreted as the excess risk attributable to vaccination. Given the nonsignificant overall association and substantial heterogeneity, the current findings do not establish a causal relationship between COVID-19 vaccination and retinal vascular occlusion. The vaccines’ track record in preventing severe COVID-19 outcomes remains well established.^[3,4]^

The unique protective features of the retinal vascular system, including the blood–retinal barrier and autoregulatory mechanisms, may explain why the vaccines were not significantly associated with the overall retinal vascular occlusion risk in our analysis, despite their capacity to transiently activate endothelium and elevate inflammatory cytokines such as Tumor necrosis factor--α and interleukin-6.^[21,30,36,37]^ These reactions typically resolve within days and appear generally insufficient to trigger localized thrombotic occlusion in most recipients.

The slightly higher vein versus artery risk observed in our analysis may reflect differential vascular susceptibility, with venous systems potentially more vulnerable to vaccine-induced inflammatory or prothrombotic states.

While our findings failed to demonstrate a statistically significant association between vaccines and overall retinal vascular occlusion, the stratified analysis of arterial and vein occlusions showed a statistically significant association; however, the absolute risk remains exceptionally low at 0.02%. The vaccines’ track record outweighs this minimal risk in preventing severe COVID-19 outcomes, saving countless lives and preventing hospitalizations across millions of recipients worldwide.

Healthcare providers should maintain awareness of potential ocular complications, particularly in high-risk populations with preexisting vascular conditions. However, these findings should not discourage vaccination.

### 4.3. Strengths and limitations

The current meta-analysis has several strengths, including its comprehensive scope, large sample size, and rigorous methodology. The inclusion of diverse geographic regions and vaccine platforms enhances generalizability. However, the substantial heterogeneity observed, likely due to the pooling of studies that differed in study region, vaccine type, vaccine dose, and follow-up durations, necessitates cautious interpretation of the pooled estimates. The revised overall analysis did not demonstrate a statistically significant association between COVID-19 vaccination and retinal vascular occlusion (RR = 2.12, 95% CI: 0.65–6.90), with substantial heterogeneity (*I*^2^ = 96.8%). Although the test for subgroup differences by follow-up duration was statistically significant (*P* = .035), several subgroups were based on individual studies, limiting the reliability of these comparisons. Accordingly, the pooled RR should be interpreted as an exploratory average across clinically heterogeneous populations and observation periods rather than a uniform vaccine-associated effect. Two pairs of studies shared potentially overlapping data sources, including the South Korean national health databases and the TriNetX research network. Although overlapping cohorts were not combined within the same pooled comparison, individual-level overlap could not be quantified. Outcome-specific study selection also reduced the number of contributing studies in several analyses, with some subgroup estimates derived from single studies. These limitations may affect the precision and generalizability of the pooled estimates. The observational nature of included studies introduces risks of confounding and selection bias, limiting causal inference. Furthermore, the pooled risk ratios were calculated directly from event counts and group totals rather than adjusted effect estimates, leaving the results susceptible to differences in baseline risk and residual confounding. The limited number of included studies also reduces the reliability of vaccine-specific and other subgroup analyses. Although potentially overlapping populations were addressed through outcome-specific study selection, the exact extent of individual-level overlap could not be determined from the published data. Finally, differences in follow-up duration limit the direct comparability of cumulative event proportions across studies.

Although the outcome-specific analyses demonstrated statistically significant associations for both RAO and RVO, these results were based on different combinations of studies than the overall analysis. In particular, the absence of Kim et al from the subtype-specific analyses may have contributed to the observed differences in statistical significance. Therefore, the significant subtype-specific findings should not be interpreted as evidence that contradicts or overrides the nonsignificant overall association.

Future studies should employ standardized case definitions, longer follow-up periods, and adjustment for potential confounders, including comorbidities, medications, and baseline ocular health. Additional large-scale studies with comparable observation periods and appropriate adjustment for baseline vascular risk factors are needed to clarify whether any independent association exists between COVID-19 vaccination and retinal vascular occlusion.

## 5. Conclusion

The current meta-analysis found that retinal vascular occlusion events were rare among COVID-19 vaccine recipients, with a pooled event proportion of 0.02%. After accounting for potentially overlapping study populations, no statistically significant overall association was observed between COVID-19 vaccination and retinal vascular occlusion. However, substantial heterogeneity and imprecise estimates limit definitive conclusions. Although exploratory subgroup analyses suggested differences by vaccine type and follow-up duration, these findings require confirmation in larger, methodologically consistent studies. Further research is warranted to clarify potential associations while recognizing the established benefits of COVID-19 vaccination.

## Author contributions

**Conceptualization:** Omar Khalid Samir, Noheir Ashraf Ibrahem Fathy Hassan.

**Data curation:** Omar Khalid Samir, Noheir Ashraf Ibrahem Fathy Hassan, Raymond Haward, Ishaan Batish, Amr Arafa, Eslam Alwani Haiba, Mahmoud Aboudabash, M. Khodr Aljamali, Mohamed AlMadhoob, Saad Yousafzai, Yusef Hazimeh, Eman A. Toraih.

**Formal analysis:** Omar Khalid Samir, Eman A. Toraih.

**Investigation:** Noheir Ashraf Ibrahem Fathy Hassan, Eman A. Toraih.

**Methodology:** Omar Khalid Samir, Noheir Ashraf Ibrahem Fathy Hassan.

**Project administration:** Hani Aiash.

**Resources:** Omar Khalid Samir.

**Software:** Omar Khalid Samir, Eman A. Toraih.

**Supervision:** Hani Aiash.

**Validation:** Omar Khalid Samir, Hani Aiash.

**Visualization:** Omar Khalid Samir.

**Writing – original draft:** Omar Khalid Samir, Noheir Ashraf Ibrahem Fathy Hassan, Eman A. Toraih.

**Writing – review & editing:** Omar Khalid Samir, Noheir Ashraf Ibrahem Fathy Hassan, Raymond Haward, Ishaan Batish, Amr Arafa, Eslam Alwani Haiba, Mahmoud Aboudabash, M. Khodr Aljamali, Mohamed AlMadhoob, Saad Yousafzai, Yusef Hazimeh, Eman A. Toraih, Hani Aiash.


















