# Supplementary material for: Risk of retinal vascular occlusion following COVID-19 vaccination: A meta-analysis of nearly 15 million individuals
Source: Medicine (Baltimore). 2026 Nov 6;105(45):e51345. doi: 10.1097/MD.0000000000051345 (PMC13747517; doi:10.1097/MD.0000000000051345)
Supplement: Supplementary file 1 [file medi-105-e51345-s001.docx]

### **Supplementary Table S1.** The details of the search strategy

| Database | Search strategy | Results |
| --- | --- | --- |
| PubMed | (COVID-19 vaccine* OR SARS-CoV-2 vaccine* OR coronavirus vaccine*  OR mRNA vaccine* OR Pfizer vaccine OR Moderna vaccine  OR AstraZeneca vaccine OR BNT162b2 OR mRNA-1273 OR ChAdOx1)  AND  (retinal vascular occlusion* OR retinal vein occlusion*  OR retinal venous occlusion* OR retinal artery occlusion*  OR retinal arterial occlusion* OR central retinal vein  OR branch retinal vein OR central retinal artery  OR branch retinal artery OR CRVO OR BRVO  OR CRAO OR BRAO OR RAO OR RVO) | 597 |
| Medline | (COVID-19 vaccine* OR SARS-CoV-2 vaccine* OR coronavirus vaccine*  OR mRNA vaccine* OR Pfizer vaccine OR Moderna vaccine  OR AstraZeneca vaccine OR BNT162b2 OR mRNA-1273 OR ChAdOx1)  AND  (retinal vascular occlusion* OR retinal vein occlusion*  OR retinal venous occlusion* OR retinal artery occlusion*  OR retinal arterial occlusion* OR central retinal vein  OR branch retinal vein OR central retinal artery  OR branch retinal artery OR CRVO OR BRVO  OR CRAO OR BRAO OR RAO OR RVO) | 105 |
| Web of Science | TS = (  (COVID-19 vaccine* OR SARS-CoV-2 vaccine* OR coronavirus vaccine*  OR mRNA vaccine* OR Pfizer vaccine OR Moderna vaccine  OR AstraZeneca vaccine OR BNT162b2 OR mRNA-1273 OR ChAdOx1)  AND  (retinal vascular occlusion* OR retinal vein occlusion*  OR retinal venous occlusion* OR retinal artery occlusion*  OR retinal arterial occlusion* OR central retinal vein  OR branch retinal vein OR central retinal artery  OR branch retinal artery OR CRVO OR BRVO  OR CRAO OR BRAO OR RAO OR RVO)  ) | 550 |
| Scopus | TITLE-ABS-KEY(  ("COVID-19 vaccine*" OR "SARS-CoV-2 vaccine*" OR "coronavirus vaccine*"  OR "mRNA vaccine*" OR "Pfizer vaccine" OR "Moderna vaccine"  OR "AstraZeneca vaccine" OR BNT162b2 OR mRNA-1273 OR ChAdOx1)  AND  ("retinal vascular occlusion*" OR "retinal vein occlusion*"  OR "retinal venous occlusion*" OR "retinal artery occlusion*"  OR "retinal arterial occlusion*" OR "central retinal vein"  OR "branch retinal vein" OR "central retinal artery"  OR "branch retinal artery" OR CRVO OR BRVO  OR CRAO OR BRAO OR RAO OR RVO)  ) | 113 |
