# Supplementary material for: Risk of retinal vascular occlusion following COVID-19 vaccination: A meta-analysis of nearly 15 million individuals
Source: Medicine (Baltimore). 2026 Nov 6;105(45):e51345. doi: 10.1097/MD.0000000000051345 (PMC13747517; doi:10.1097/MD.0000000000051345)
Supplement: Supplementary file 2 [file medi-105-e51345-s002.docx]

**Supplementary Table S2:** Quality assessment of the included studies.

| **Study** | **S1** | **S2** | **S3** | **S4** | **C1** | **C2** | **E1** | **E2** | **E3** | **Total** |
| --- | --- | --- | --- | --- | --- | --- | --- | --- | --- | --- |
| Park 2024 | 1 | 1 | 1 | 1 | 1 | 1 | 1 | 1 | 0 | 8 |
| Kim 2024 | 1 | 1 | 1 | 1 | 1 | 1 | 1 | 1 | 0 | 8 |
| Li 2023 | 1 | 1 | 1 | 1 | 1 | 0 | 1 | 1 | 0 | 7 |
| Dorney 2023 | 1 | 1 | 1 | 1 | 0 | 0 | 1 | 0 | 0 | 5 |
| Hashimoto 2022 | 1 | 1 | 1 | 1 | 1 | 1 | 1 | 0 | 0 | 7 |

The Newcastle-Ottawa Scale assessment criteria are as follows: Selection criteria (S1-S4) evaluate the representativeness of the cohort (S1), selection of controls (S2), exposure ascertainment (S3), and demonstration that the outcome was not present at the start (S4). Comparability criteria (C1-C2) assess control for age/sex (C1) and additional factors (C2). Exposure criteria (E1-E3) examine the assessment method (E1), whether the follow-up was long enough for outcomes to occur (>8 weeks) (E2), and adequacy of follow-up (E3). Each criterion is scored as met (1) or not met (0), with a maximum possible total score of 9 points.
